# The role of the African value of Ubuntu in global AI inclusion discourse: A normative ethics perspective

**DOI:** 10.1016/j.patter.2022.100462

**Published:** 2022-04-08

**Authors:** Arthur Gwagwa, Emre Kazim, Airlie Hilliard

**Affiliations:** 1 Ethics Institute, Department of Philosophy & Religious Studies, Utrecht University, Janskerkhof 13, 3512 BL Utrecht, The Netherlands; 2 Department of Computer Science, University College London, Gower Street, London WC1E 6EA, UK; 3 Holistic AI, 18 Soho Square, London W1D 3QH, UK; 4 Institute of Management Studies, Goldsmiths, University of London, New Cross, London SE14 6NW, UK

**Keywords:** African ethics and values, artificial intelligence, AI ethics, Ubuntu, utilitarianism

## Abstract

Historically, Sub-Saharan Africa (SSA) has been excluded from the benefits of the previous industrial revolutions, as its people and their resources and aspirations have been objectified through foreign domination, and its culture has either been fragmented or appropriated. While artificial intelligence (AI) is poised to generate vast amounts of wealth, its application could lead to further social and economic exclusion of SSA due to a lack of access to technological advancements and the historical injustice and exclusion based on protected characteristics. Through an examination of the concept of inclusion, this paper explores how to improve the terms on which African populations and subpopulations and their concerns are included in the global AI ethics discourses. Specifically, it is argued that the SSA value of Ubuntu could be of immense value in AI applied normative ethics, particularly toward an inclusive approach for the implementation of the universal AI ethics principles and guidelines.

## The bigger picture

AI’s social and economic benefits and its challenges to the African social and cultural perspectives are beginning to catch the attention of African policy makers if judged by the proliferation of AI think tanks and empirically grounded policy recommendations. However, unlike other regional blocks, like the EU, unified African positions and approaches in global AI ethics forums remain sparse. Although the current universal ethical guidelines and principles can provide Africa with a common ground with other cultures, care is needed in localizing these principles, as they may not be applicable in the African context.

The global ethics discourse can capitalize on the emerging African ethical relational approaches, underpinned by Ubuntu, to devise frameworks that would assist the implementation of the universal values, such as justice and solidarity, in a manner that pays regard to cultural environments of historically marginalized populations, like in Africa. Future research and policy work should ideally focus on coming up with Ubuntu-based action guiding principles for all AI stakeholders. These could, for example, include guidance on reconciling competing and often conflicting cultural values and ethical dilemmas in AI design, development, and audits. This and related works can only have impact if forums are created for interdisciplinary discourses between policy makers, technologists, ethicists, and philosophers to ensure that the African context is being considered in their work.

## Teaser

While AI is poised to generate vast amounts of wealth, its application could lead to further social and economic exclusion of Sub-Saharan Africa (SSA), mostly due to the historical injustice and exclusion. The SSA value of Ubuntu can provide an ethical base for the inclusion discourse. In particular, its emerging views on its relational autonomy approach, by bridging the Global North and South ethical perspectives, can aid an inclusive approach for the implementation of the universal AI ethics principles and guidelines.

## Introduction

While there is a consensus about the enormous potential for artificial intelligence (AI) to advance development and solve some of the most pressing challenges faced by Sub-Saharan Africa (SSA), discussions of the ethical challenges that AI will bring to Africa have only just begun. Little has been done to advance unified African positions and approaches in global AI ethics forums. This is despite the rise in recent literature on how one might apply AI to resolve problems in Africa and on ethical issues facing AI’s application to Africa, particularly “the need to define African values and align AI with them.”^1^ Nevertheless, some think tanks have emerged and are producing empirically grounded policy recommendations. However, while policy has a role to play, there is very little examination of fundamental issues relating to the values underpinning such policies and, in particular, how to address the AI risks and challenges that may be more acute in the Global South, where the low access to AI technology could lead to exclusion, particularly in SSA. In the past decade, there has been an emergence of notable works with mainstream African ethical approaches, with some addressing the need for African relational approaches in addressing AI algorithms injustices,^2^ balancing relational approaches with autonomy,^3^ and explicability.^4^ By building on these emerging views, this paper argues that at the heart of Ubuntu are principles that prescribe the virtues needed, procedures, and the desired consequences in the application of universal AI ethical principles. This would lead in the systematic integration of the universal AI principles and an inclusive deployment of AI technologies. By seeing one’s humanity in the humanity of others, Ubuntu resonates with the golden rule that cuts across major world cultures: We should do to others what we would want others to do to us. Further, relying on a value from SSA—generally the world’s most economically disadvantaged region—would be of a practical and symbolic benefit use toward the greater inclusion of SSA. While Ubuntu’s relational approach based on communitarianism is not unique to Africa, it would be of practical and symbolic benefit toward greater inclusion of SSA in AI ethics discourse and the economic and social benefits resulting from AI, particularly because it widely informs most African subcultures and looms large in the SSA philosophy and ethics.^5^

SSA’s exclusion resulting from the deployment of AI has the potential to both perpetuate and amplify the deep-rooted exclusion of Africans for three key reasons, with exclusion referring to “the inability to participate effectively in economic, social, political, and cultural life, and, in some characterizations, alienation and distance from the mainstream society.”^6^ Firstly, AI can amplify or reinforce long-standing societal biases, particularly those related to characteristics protected under international human rights law, such as race and culture. Secondly, as Africans can lack the capacity to access and apply their data, they are less able to develop and implement AI and so miss out on the economic benefits it can bring. Finally, since it is predominantly the perspective of the Global North that is informing the current discussions on inclusion, in certain circumstances, this has resulted in weak commitment to addressing historical social and economic injustices. While a plethora of guidelines on ethical or responsible usage of AI is emerging, each promotes different values and definitions,^7^ meaning that care is needed when drawing on generic principles that may or may not be universal in scope.^4^ This includes paying attention to the social, cultural, and local values of the region in which these principles are being applied; Africa has historically seen misaligned foreign values imposed on it as a result of these factors not being considered.^7^

In this paper, we therefore argue that the relational SSA philosophy of Ubuntu, which emphasizes one’s personhood to the personhood of others, could be of both normative and applied practical value toward the realization of the current corpus of principles and guidelines on ethical AI. As shall be demonstrated below, the values that are being currently appealed to in AI ethical discussions, such as solidarity and those based on medical ethics,^8^ including autonomy, justice, beneficence, and non-maleficence, can only bring utility if there are generally agreed upon adequate implementation strategies.^9^ The multiple cultural contexts in which AI is applied may present a barrier in the even application of these principles and guidelines in a manner that ensures an equitable distribution of AI benefits across the globe. Adopting the value of Ubuntu does not just acknowledge a unique contribution by Africans to general philosophy and applied ethics but gives guidance on the virtues, procedures, and desired consequences toward an inclusive and ethical AI. As an example, Ubuntu reinforces the universal value of solidarity to the extent that it proposes communal relations based on generosity, hospitality, compassion, and friendliness.^10^^,^^11^ We argue that such characteristics of Ubuntu values are at the center of what it means to be human in a world with competing and often conflicting cultural values.

## AI ethics and exclusion challenges

Historically, SSA has been excluded from the benefits of the previous industrial revolutions, as its people and their resources and aspirations have been objectified through slavery, colonialism, imperialism, and neo-colonialism. While the slave trade was meant to exploit African resources to feed the ever-expanding European markets, today, African resources are again powering the Fourth Industrial Revolution. From the Congolese cobalt crucial for the manufacturing of computer chips to the data that are being used to train AI algorithms, African resources are significantly shaping the future of AI. However, just like in the previous industrial revolutions, African voices are absent from shaping the future of these developments. As the Fourth Industrial Revolution progresses, it is therefore important to assess the extent to which SSA, in all its diversity and similarities, is being included in the discussions and benefiting from the outcomes of the various social, economic, and political systems and processes underpinning the current changes. In this context, inclusion refers to the process of improving the terms on which individuals and groups can take part in society and the ability, opportunity, and dignity of those disadvantaged on the basis of their identity.^12^ The United Nations has emphasized the importance of inclusion in a number of their sustainable development goals,^13^ claiming that this systematic process can rescue a person or community from the risks or uncertainty of exclusion.

### Exclusion at the continental level

While SSA is made up of a diverse range of countries, they share broad similarities, like their history; aspirations, which are mostly shaped by the liberation wars, past political junctures, and trajectories; and a broadly similar communitarian cultural value system ensuring an appropriate ethical and legal framework to strengthen African values. An additional attribute shared by these countries is that they historically have not benefited or have been excluded from the benefits of the previous industrial revolutions. With the onset of the Fourth Industrial Revolution mostly underpinned by AI, Africans may be excluded from the benefits of AI on the grounds of natural characteristics or protected attributes, including color, language, culture, or race, as a result of the limited or unrepresentative African datasets available for the proper training and application of algorithms or AI applications, like facial recognition software. Since the AI field is mainly composed of white males, this lack of diversity and inclusion has already resulted in flawed systems that amplify gender and racial biases, according to a survey carried by the AI Now Institute, which examines the social implications of artificial intelligence.^14^ “The media is filled with unintended ethical concerns of AI algorithms, such as image recognition algorithms not recognizing persons of color or racist algorithmic predictions of whether offenders will recidivate.”^7^ Calls to correct anomalies and flawed systems have sometimes been received unkindly by technology firms, as was evident in the dismissal of Timnit Gebru, co-leader of Google’s Ethical AI team, who surfaced the dangers of large language models like the ones that power the company’s search engine.^15^

Given that AI stands to generate vast wealth for the corporations and countries that develop it, the rest of the world could be left behind if they are excluded from the social, cultural, and economical benefits of AI.^16^ It is, therefore, evident that there needs to be an effort toward greater inclusion in this domain, particularly since the Global North lacks the insight needed to create solidarity in these advancements. First, this is due to the disconnect between the algorithm designers and the communities where the research is conducted or algorithms are implemented.^7^ Secondly, governance, including in the AI domain, in liberal democracies of the Global North is mainly focused on protecting autonomy within the individual private sphere.^17^ This is a typical Western worldview that centralizes the individual and which is reflected in bioethical principles, like the principle of respect for autonomy, frequently understood as respecting the decisional autonomy of an individual who makes decisions without undue coercion.^18^ Consequently, there is an acute need for increased and organic interactions among intellectuals globally to facilitate the expansion of this discourse beyond the Western world, particularly because the reality of global exclusion is felt most in the developing world.^19^

### Exclusion at the national level

Global corporations, including those working on technology and data, are involved in data-mining activities in Africa that are not just amplifying existing societal tensions but also excluding African subpopulations who represent low-value data.^20^ This exclusion is also seen in the uneven access to data, AI, and related technologies, as well as the impact of these tools, which is greatest in marginalized populations.^21^ This impact is particularly felt in the least developed countries, who sit at the intersection of these marginalized groups, resulting in the amplification of these digital inequalities across the world. Non-representative or biased data can further entrench existing inequities as AI systems reflect the biases and lack of representation of the datasets on which they are trained, resulting in the exacerbation of the long-standing societal biases that exist surrounding protected characteristics, like race.^22^ Data are expensive and hard to come by at scale, but the data that are available encompass three broad groups of people: the uncounted who do not exist because they are not included in any sort of database; the unaccounted who have less inclusion into the digital world and therefore not entirely represented, maybe due to economic reasons; and the discounted who are in databases but are not of interest to the people who would serve them, such as governments or companies, because they do not have enough money to be of concern. AI algorithms are trained on the data that are available, as opposed to complete datasets, and these data can easily privilege socio-economically advantaged populations who have greater access to connected devices and online services.^23^ As a result, the populations who do not have this access are often forgotten and the gap between developed and undeveloped countries widens. Therefore, initiatives are needed to increase the fairness and representativeness of data and algorithms and an examination of the values that they embody to facilitate greater inclusion. In support of this, African scholars are beginning to explore sociological approaches that go above and beyond technical solutions by placing ethics in their “relational” context^2^ and how to reconcile relational approaches with autonomy.^3^

## Do current initiatives embody African values?

There has been a steady increase in the number of global and regional AI ethics initiatives that have by-and-large been aimed at addressing the kinds of exclusions discussed in AI ethics and exclusion challenges. It is also common for them to include the rights of persons at risk of exclusion, improving the individual and collective wellbeing and dignity of these people and allowing them to flourish.^24^ As an example, in their comprehensive map of the corpus of principles and guidelines on ethical AI, Jobin et al.^9^ reveal a global convergence emerging around five ethical principles (transparency, justice and fairness, non-maleficence, responsibility, and privacy). Nevertheless, they also report a “substantive divergence in relation to how these principles are interpreted; why they are deemed important; what issue, domain or actors they pertain to; and how they should be implemented,” thus highlighting the importance of the applicability and the question of implementation of these principles in different contexts.^9^ Carman and Rossman^4^ call attention to the need for care when drawing on generic principles that may or may not be universal in scope, including by paying attention to the cultural context, especially in post-colonial Africa, given its history of the imposition of external values. Despite claims of universality, most AI ethics principles and their guidelines are developed by stakeholders based in economically developed, mostly Western countries, like the United States and from within the European Union.^9^ As a result, some aspects of the principles may not automatically apply in Africa without the necessary adjustments. For example, the principle of respect for autonomy may be incompatible with the African communitarian approach to decision making.^4^ Yet a common ground can be found if it is based on the idea of personhood in African traditions, which imply “a relational and positive sense of autonomy, which involves the community helping or guiding one to use one’s ability and knowledge of one’s social relations and circumstance to choose freely the requisite goods for achieving one’s life plan.”^3^

The current exclusion of Africa, including its ethical approaches to AI governance, whether intentional or unintentional, means the inclusion debate is still framed from the perspective of the Global North, who developed the technology in accordance with Western perspectives, values, and interests with little regulation or critical scrutiny.^25^ As African and South-American countries are not represented independently from the international or supra-national organizations that are producing these guidelines, this may present a barrier to implementation of such guidelines but also the deployment of the AI technologies in specific sectors, such as agriculture, where, for example, excessive automation may disrupt the African way of life that revolve around certain customs.^26^

In addition, the private-sector companies from the developed countries have been involved in the AI-ethics arena, thus raising concerns that they may potentially use such high-level soft policy as a portmanteau to either render a social problem technical or to eschew regulation altogether.^9^ Given the non-inclusion of stakeholders from Africa and South America, the convergence of AI ethics set of principles on the four classic principles of medical ethics, namely autonomy, justice, beneficence, and non-maleficence, will not address Africa’s concerns about inclusion, as the implementation of these high-level principles can conceal deep political and normative disagreement, which could have unwanted effects on the future of AI development and governance.^27^ As an example, while the European AI4People’s recent publication^28^ interprets justice to include using AI to right previous wrongs, ensuring that the benefits of AI are shared fairly, the wealth from AI still benefits a few developed countries that unfairly benefitted from the previous industrial revolutions. The justice articulated in the ethics discourse should be accompanied by implementation guidelines on how to specifically include historically marginalized populations whose resources were used to power the previous and continue to power the current industrial revolutions. As suggested by the United Nations Educational, Scientific and Cultural Organization (UNESCO), the global AI ethics initiatives should frame Africa as a cross-cutting concern.^29^ Ethics do not just influence human decisions on what is right or wrong but constitutes the basis of future action, and in the case of AI, it will influence the course of the Fourth Industrial Revolution. Under such circumstances, a relational approach to ethics may be more sensitive to the African cultural context, since it advances the notion of inclusion. Best practices toward inclusion can be seen in other cultures and domains, such as the way Canada acknowledges the historical injustices to the First Nations, particularly in land ownership. Similarly, Africans should explicitly be asked how they want to be included in the revolution and on what terms. Colonization dispossessed Africans of more than resources and self-governance; it also took their voice, ability to self-determine, collective agency—the ability to negotiate with a unified voice, and, in some instances, appropriated African culture.^30^ Ironically, the colonizers did not appropriate the essence of African culture captured in Ubuntu, but inclusion of this value in the Fourth Industrial Revolution would be an important step in implementing the converging global AI substantive values.

### Reshaping the Western concept of inclusion

So far, much of the literature and research on social exclusion is underpinned by frameworks that are concerned with European and Anglo-Saxon traditions. As such, they ignore the contributions made by people of Africa, Asia, and Latin America, where global exclusion is more likely to be felt. A second challenge is the marked absence of any discussion on power embedded in social relations and the disruption of relationships between individuals and society.^12^ Consequently, the European and Western model for inclusion in AI and technology in general should be rearticulated to draw on input from the Global South and create a more developmental focus on global inequalities.

The future of the inclusion debate will depend on the ability to develop a global inclusion initiative that draws on the intellectual capacities of both the Global North and the Global South.^12^ Specifically, Africans should define what inclusion means to them and how it can be achieved, since there is only a tepid commitment to addressing historical injustices, like how African people and their resources and aspirations have been objectified through slavery, colonialism, imperialism, and neo-colonialism. These injustices are still relevant in the AI era, which is creating new domination capabilities and novel problems; while traditional colonialism is driven by political and government forces, algorithmic colonialism is driven by corporate agendas.^25^ In Kenya, for example, AI and data-optimization technologies are exploiting existing ethnic and racial tensions, particularly during election times, through computational hate propaganda and disinformation.^31^ These technologies are undermining the basic values of African societies, such as community, but also the concepts that are characteristic of African normative ethical thinking, including harmony, consensus, collective action, and common good. The effectiveness of this discourse could be maximized if it were adapted to cultural or country-specific situations where codes could potentially have policy relevance.^12^ In addition, as Timnit Gebru and her colleagues attempted to champion, there should also be more comprehensive action against racism, sexism, and other forms of socially constructed exclusions, something which has been lacking in past discourses but is beginning to emerge in the Africa AI decolonization movement.^25^

### Emerging African views

Although AI ethics guidelines and principles and their accompanying industrial codes of ethics and toolkits are a good starting point, they alone cannot resolve the disparities highlighted above without respectful and honest dialogue between the two hemispheres to address the historical disadvantage and value misalignment whereby AI reflects Western values, agendas, and motives. So far, the idea or willingness to find universal principles is neither healthy nor efficient, given the exclusion of billions of people from participating in the framing of these principles that will affect them and their future generations. However, efforts toward these dialogues have already proven useful, particularly the workshops of the UN Global Pulse, which were held in Ghana and Tunisia.^32^ From these workshops emerged a unanimous consensus that Africa could learn from the Global North’s mistakes to ensure that they do not develop technologies without first formulating a set of values to guide them. In addition, Africans advocated for the need for human control of technology and the promotion of human values, something which has been reactionary rather than proactive in global principles.^33^

From these workshops, which the first author attended, emerged some key principles:

#### The need to define African values and align AI

An important point raised during the Ghana workshop was that African countries should clearly define and refine their own ethical values to allow effective regulations and policies to be introduced that are reflective of the values of the specific cultural and religious contexts in which they are applied. This would allow African countries to adapt global initiatives to align with values like Ubuntu, which encompass a collective approach to life. However, the task of value alignment, namely, how to align AI with human values, faces some challenges. Since it is argued that Africa is not homogeneous, it is questioned which “African” values should be embedded in algorithms that are applied throughout Africa.^33^ Even at a policy level, unlike the collective policy response to emerging technologies that is seen in Europe, the African Union does not have an effective rule-making mechanism, so each African country, with its own peculiarities, is tasked with its own rule making.^34^ There are also differences between Africa and the Western world at a philosophical level. For example, while the consequentialist conception of utilitarianism guides much thought about ethics and public policy in the 21^st^ century,^1^ Metz argues that “utilitarianism prescribes a number of immoral actions in the light of some plausible beliefs common in African ethical thought, and supposing that moral actions are necessarily rational ones, these criticisms also implicitly cast doubt on the apparent rationality of utilitarianism.” This view is backed by Mhlambi, who asserts that AI is based on the Western conception of rationality, which excludes and discriminates against those who do not measure up to it.^35^ African philosophers are fragmented by the neoliberal divisive agenda that has been used to ensure Africa does not have a unified philosophical approach and collective rule making by insisting Africa is not homogeneous. Unlike post-modernist scholars on pan-Africanism who hold such a divisive view, Africanist scholars argue for the importance of collective identity in the struggles of people of African descent.^36^ SSA can obtain inspiration to work communally from the sense of solidarity exhibited by the African diaspora in their struggle for civil rights, which has become an important source of self-esteem and political strength for African Americans.^27^^,^^35^

#### The significance of Ubuntu as a universal African value

In Africa, or at least in southern Africa, the Zulu term Ubuntu (“a person is a person through other persons”) has been used to describe African morality and way of life. This maxim is echoed by John Mbiti, “Whatever happens to the individual happens to the whole group, and whatever happens to the whole group happens to the individual. The individual can only say: ‘I am, because we are and since we are, therefore I am.’^37^ Ubuntu has been further expanded by African philosophers to qualify as a moral theory that has led to various interrelated concepts, including the need for an individual to subject themselves to their community to qualify for personhood. In this sense, a communitarian social arrangement defines African culture and characterizes social relations among individuals in African societies.^38^ Even before the emergence of philosophy as a distinct discipline in Africa, there were numerous case examples in Africa where such overarching moral principles have been used to resolve difficult moral decisions, as documented in traditional Ashanti consensual political culture.^39^ There are also early examples of how Africans welcomed Europeans as part of their community, with Zimbabwe’s spirit medium Nehanda Charwe Nyakasikana being known to promote good relationships between the Zezuru people and European settlers. In his work, Ikuenobe makes references to some African traditions to illustrate how the African communitarian conception of personhood and autonomy was a recurring theme in African cultures.^3^

By coming up with their own moral theories, African ethicists seem to be rejecting the Western concept of utilitarianism, which aims to construct AI that maximizes what is good for human beings and minimizes what is bad for them in the long run.^40^ In doing so, they have made further progress in articulating African cultural values and customs, such as the collective and communal approach to life and work, and integrating these in technological implementation as part of their reflective turn in the ethics of technology.^1^ Through this application of Ubuntu, there is a drive toward greater inclusion and diversity in the global AI ethics discourse, particularly the inclusion of African voices.^41^

However, despite the touting of Ubuntu as an African moral philosophy, questions still remain on its application in normative ethics and how it sits with universal values and their application in AI ethics. For instance, if the current corpus of universal values speaks to all or some of the African concerns, should Ubuntu replace or reinforce such values, and if so, how? As has been discussed above, Ubuntu could help in devising frameworks that would assist the implementation of the universal values, such as justice and solidarity, in a manner that pays regard to cultural environments of historically marginalized populations, like in Africa. Ubuntu can bridge the gap between theoretical principles and applied local contexts to the extent that it reinforces the virtues, procedures, and desired outcomes, such as communitarianism, which can constitute both a virtue, procedure, and an outcome. By seeking the common humanity of human beings, Ubuntu can help reconcile competing and often conflicting cultural values, creating a sense of solidarity through global communal relations based on generosity, hospitality, compassion, and friendliness.^10^^,^^11^

### Justifying Ubuntu as an ethical base for the inclusion discourse

A collective rights approach to AI ethics could be beneficial to the rest of the world if Africa’s Ubuntu ethics and the normative principles emerging from it are incorporated into the global AI ethics discourse. Doing so would mean that technology would be more reflective of the value of life or communion and communal relationships, which are characterized by identification with others and exhibition of solidarity with them.^1^ The African approach also values a sense of togetherness and cooperative participation, which could lead to the inclusion of Africans in defining ethical standards.

Ubuntu is not just a basis for communities but can also be a basis for the inclusion discourse and be something Africa can export to global forums in proposing how the benefits of AI can be shared. As the Suthu and Nguni philosophers argue, Ubuntu is one of Africa’s greatest gifts to the world.^35^ Evidently, there is a significant role that African philosophers can play in developing new theories and methods that are necessary to understand, morally assess, and intervene in the development and implementation of AI. This would promote the inclusion of values like harmony, consensus, collective action, and common good, which are characteristic of African normative ethics, in the global discourse and AI policy.

## Conclusion

While technology is increasingly produced, marketed, and used by people and organizations with a non-Western background and ethical issues concerning technology increasingly involve intercultural encounters,^42^^,^^43^ there is still a lack of inclusion of countries from the Global South in the discourse surrounding the ethical use of AI. This lack of consultation of less developed countries is particularly significant, since they are more likely to feel the negative impacts of AI. Efforts toward the necessary dialogue between the Global North and South have identified the need for further action toward greater inclusion of underrepresented continents like Africa, with African countries being encouraged to define their values and apply them to policy. Greater representation of these values may not only be a means to respond to AI’s disproportionate negative effect on people but also to achieve global equality and protections from the bottom up^35^ by promoting solidarity and a sense of togetherness.

African values like Ubuntu, as well as the proposed moral ethics principles like harmony and consensus, have the potential to have a significant influence on AI ethics and policy, but this is only possible if the current domination of discourse by the Global North ceases. Greater inclusion would result in AI being more accessible and having less adverse effects for marginalized populations. A reflective turn in the ethics of technology is, therefore, necessary, and it should draw on a conception of ethics that encompasses broader social and political themes. Such an approach that broadens the ethics discourse^44^ would allow the psychological, social, and political impact of emerging technologies to be assessed, potentially narrowing the gap between the access of AI in developed and less developed countries.
